# Fish bone foreign body causing rectal, uterine, and bladder perforation: a rare case report

**DOI:** 10.3389/fonc.2025.1768218

**Published:** 2026-01-14

**Authors:** Shiyu Luo, Bo Gou, Tengyu Zeng, Jian Liu, Jun Li, Jiangying Zhou

**Affiliations:** 1 Department of Ultrasound, The First Affiliated Hospital of Chengdu Medical College, Chengdu, China; 2 Department of Ultrasound, The First People’s Hospital of Jintang County, Jintang, China

**Keywords:** bladder perforation, fish bone foreign body, imaging examination, rectal perforation, uterine perforation

## Abstract

Fish bones are one of the most common gastrointestinal foreign bodies resulting from accidental ingestion. While the majority can pass through the gastrointestinal tract via peristalsis, a small proportion may become lodged in the digestive tract, potentially causing injury to the gastrointestinal wall or adjacent tissues. However, visceral perforation remains extremely rare. Preoperative abdominal imaging examinations provide crucial evidence for the diagnosis and localization of fish bone foreign bodies. This article reports a case of a patient presenting with painless hematuria who was initially suspected to have malignant bladder lesions. Through multimodal joint analysis, the diagnosis of foreign body-induced bladder perforation was established, which was subsequently confirmed by Collaborative Laparoscopic Therapy to be caused by a fish bone perforating the rectum, uterine isthmus serosa, and bladder, accompanied by the formation of surrounding inflammatory granulation tissue. Due to the extreme rarity of fish bones becoming lodged in the lower gastrointestinal tract and causing perforation of multiple organs, this case is being reported.

## Introduction

Fish bone-induced gastrointestinal injuries are predominantly localized to the upper digestive tract, typically manifesting as foreign body sensation, dysphagia, and localized mucosal damage (1, 2). In rare cases, they may migrate to the lower gastrointestinal tract with peristalsis (3, 4), causing intestinal perforation and leading to localized inflammatory changes or abscess formation (5). Previous reports indicate that fish bone injuries are mostly confined to extraluminal spaces without penetrating adjacent organs (6), with only Cho MK et al (7). reporting one case of rectal perforation with pelvic abscess formation caused by a fish bone that exhibited migratory behavior during treatment and ultimately resulted in bladder perforation. The current case describes a fish bone located at the pelvic peritoneal reflection in a female patient that directly penetrated the rectum, bladder posterior wall, and uterine isthmus serosa, representing an exceptionally rare occurrence.

## Case presentation

A 55-year-old female patient was admitted to the hospital due to “painless hematuria for six months, worsening for two days.” Over the past six months, the patient had occasionally experienced hematuria but had not received any treatment. Two days prior to admission, the patient developed accompanied by total gross hematuria by urinary frequency and urgency after abdominal straining, with symptoms gradually worsening, prompting her to seek medical attention at our hospital. The patient has a history of hypothyroidism for over 10 years and hypertension for over 20 years, both well-controlled with long-term oral medication. The patient denies any history of surgery or a family history of hereditary tumors.

**Figure 1:**
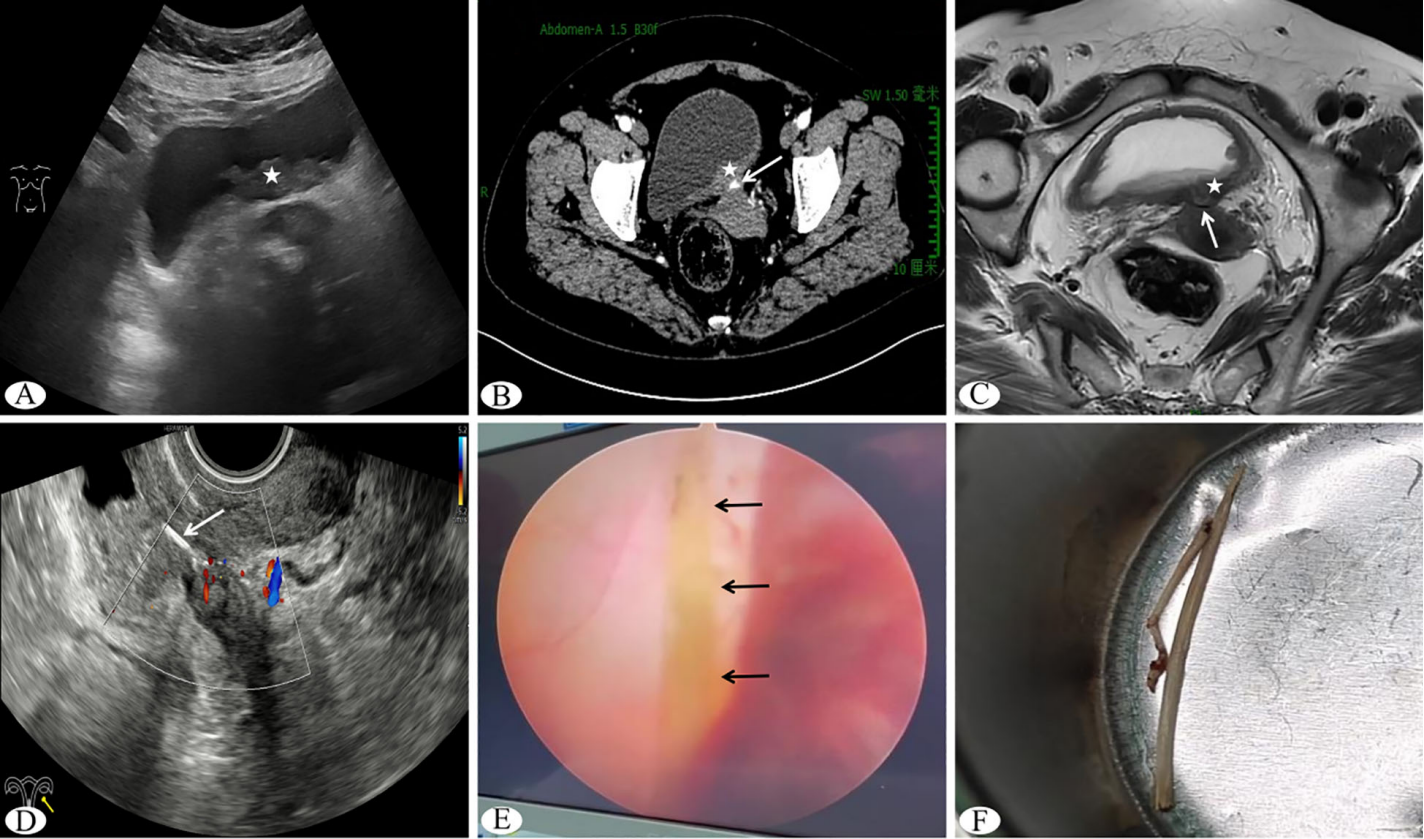
**(A)** Transabdominal ultrasound showing a posterior bladder wall mass (indicated by pentagram); **(B)** Contrast-enhanced CT demonstrating posterior bladder wall mass (pentagram) and linear dense shadow (arrow); **(C)** Contrast-enhanced MRI showing posterior bladder wall mass (pentagram) and linear hypointense signal (arrow); **(D)** Transvaginal ultrasound revealing a linear hyperechoic band in the uterovesical space (arrow); **(E)** Laparoscopic view of fish bone (arrow); **(F)** Extracted fish bone postoperatively, measuring approximately 3 cm in length.

Abdominal ultrasound examination revealed irregular thickening of the posterior bladder wall, measuring approximately 5.3×1.9×5.5 cm, with punctate abundant blood flow signals on color Doppler flow imaging (CDFI), suggestive of malignant neoplastic lesions (**Figure 1A**). Laboratory tests: White blood cell count 7.14 × 10^9^/L, Neutrophils 4.08 × 10^9^/L, C-reactive protein 2.0mg/L, Liver and kidney function tests and tumor markers (—).The urology department performed a “transurethral bladder electroresection biopsy,” during which a broad-based neoplasm with cauliflower-like growth pattern, measuring approximately 5.0×2.0 cm, was observed on the posterior bladder wall, exhibiting rich vascularity at its base with accompanying hemorrhage. Postoperative pathology indicated chronic inflammatory changes of the bladder mucosa with submucosal inflammatory granulation tissue formation. Three days postoperatively, the patient experienced recurrent hematuria. Contrast-enhanced computed tomography (CT) demonstrated irregular thickening of the posterior bladder wall with moderate persistent enhancement, showing ill-defined borders with the uterine isthmus and a linear dense shadow of undetermined nature between them (**Figure 1B**). Cervical liquid-based cytology revealed negative for intraepithelial lesion or malignancy, with reactive cellular changes associated with inflammation. Pelvic contrast-enhanced magnetic resonance imaging (MRI) revealed significant thickening of the posterior bladder wall with soft tissue signal nodularity, blurred fat planes around the posterior bladder wall, and indistinct borders with the anterior wall of the uterine isthmus, where a linear hypointense signal was noted without enhancement on post-contrast images, suggesting either post-biopsy changes or foreign body (**Figure 1C**). Subsequent transvaginal ultrasound examination showed obliteration of the uterovesical space with a hypoechoic mass measuring approximately 4.5×2.3×1.9 cm exhibiting irregular morphology and indistinct borders with both the posterior bladder wall and uterine isthmus. Within the mass, a linear hyperechoic band was seen extending continuously from the uterine isthmus into the thickened posterior bladder wall without significant posterior acoustic shadowing (**Figure 1D**). CDFI demonstrated relatively abundant punctate and linear blood flow signals within both the hypoechoic mass and the posterior bladder wall. Ultrasound findings suggested an inflammatory mass in the uterovesical space, with the internal hyperechoic band indicative of a foreign body.

Based on the aforementioned findings, the patient underwent “laparoscopic combined with cystoscopic foreign body removal.” Intraoperatively, a hard mass measuring approximately 5×3 cm was found at the pelvic peritoneal reflection, surrounded by inflammatory hyperemia and densely adherent to the rectal wall, posterior bladder wall, and uterine isthmus. Upon dissecting the mass, a linear foreign body was identified with one end originating from the rectal wall and the other penetrating through the bladder muscular layer, while one side was embedded in the uterine isthmus serosa (**Figure 1E**). The foreign body and mass were subsequently removed via combined cystoscopy, confirming the foreign body to be a fish bone (**Figure 1F**). Postoperative pathological examination of the mass revealed reactive inflammatory cellular changes without evidence of malignant cells.

## Discussion

Following accidental ingestion, most fish bones become lodged in the pharynx or esophagus, while a smaller portion reaches the stomach where gastric juice may partially dissolve them, causing smaller fragments to soften, break down, and eventually pass through the intestines. Larger fragments may resist complete decomposition, retaining their sharp edges, and when they enter the lower gastrointestinal tract, intestinal peristalsis may fail to fragment them while increasing the risk of intestinal wall perforation. On one hand, fish bones can cause localized mucosal damage leading to ulceration and perforation; on the other hand, they may penetrate the intestinal wall, causing peritonitis, abscess formation, inflammatory pseudotumors, and other complications. In rare instances, fish bone migration may occur, damaging organs such as the liver and pancreas (8), with physical examination typically revealing abdominal tenderness and rebound tenderness. The index patient’s initial presentation consisted of painless gross hematuria with a prolonged clinical course, while the typical signs and symptoms of gastrointestinal fish bone foreign body were notably absent. Meanwhile, as the condition progressed over time, the fish bone penetrated the rectum, uterus, and bladder, progressively irritating surrounding tissues and inducing inflammatory reactions and stromal proliferation, ultimately forming a granulomatous encapsulation around the foreign body. These pathological changes resulted in complex imaging manifestations that were easily mistaken for neoplastic lesions (9). Previous literature has reported a case where a migrating fish bone caused a liver abscess that was misdiagnosed as hepatic metastatic tumor (10). Given the nonspecific clinical presentation and initial imaging findings in our patient, the condition was readily misdiagnosed as a neoplastic lesion.

The patient was admitted with painless total gross hematuria, the initial imaging modality employed was abdominal ultrasound, which revealed a bladder mass with rich vascularity and poorly defined borders with adjacent organs, leading to the initial suspicion was for a malignant bladder tumor. However, laboratory tests revealed normal tumor marker levels. Therefore, transurethral resection of the bladder with biopsy was performed. Postoperative pathological examination confirmed chronic inflammatory changes of the bladder mucosa accompanied by submucosal inflammatory granulation tissue formation. These findings indicate a state of cellular instability in the bladder mucosa, which is likely attributable to inflammatory stimulation. However, the possibility of secondary involvement from adjacent organ pathology cannot be entirely excluded. Furthermore, pelvic CT revealed an ill-defined border between the lesion and the uterus. Consequently, a collaborative gynecological examination was conducted to rule out potential cervical pathology. Further multimodal imaging was performed to delineate the lesion’s intrinsic characteristics and its anatomical relationships. This ultimately led to the discovery of a foreign body, which was surgically confirmed to be a fish bone that had perforated the rectum, uterine isthmus serosa, and bladder. Retrospective analysis indicates that the clinical presentation strongly suggested a malignant lesion of urinary origin, and the initial imaging workup only included transabdominal ultrasound, which primarily focused on intraluminal bladder pathology while providing limited visualization of extravesical structures. this led to the initial misdiagnosis of primary bladder malignancy. This case demonstrates that preconceived notions based on clinical symptoms can readily divert the diagnostic process. Furthermore, the importance of not only carefully analyzing the sonographic features of the primary lesion during ultrasound examinations but also paying close attention to extra-organ imaging characteristics, particularly when lesion borders with adjacent structures are indistinct. Secondly, this case underwent a combined analysis of multiple findings following biopsy, progressively ruling out the possibility of malignant tumors originating from the bladder or its adjacent structures. Based on this, multimodal imaging studies revealed a linear structure within the lesion. This structure appeared as hyperdense on CT, hypointense on MRI, and hyperechoic on ultrasound. This combination of features points to a substance that appears hard, dense, and acoustically resistant on imaging, such as osseous tissue, calculi, or metallic objects. Moreover, the continuous and regular morphology of this structure suggests an abnormally located bony or metallic foreign body rather than intrinsic tumoral calcifications. This case clearly demonstrates that for complex lesions of uncertain etiology that challenge a single specialty, organizing a multidisciplinary team to conduct an interdisciplinary analysis holds significant clinical value in achieving a precise diagnosis.

Regarding the management of gastrointestinal fish bone foreign bodies, the European Society of Gastrointestinal Endoscopy guidelines recommend that in adults with retained gastrointestinal foreign bodies, endoscopic treatment modalities—including esophagoscopy, gastroduodenoscopy, colonoscopy, cystoscopy, ureteroscopy, and laparoscopy—should be selected based on comprehensive evaluation of clinical symptoms, general condition, and imaging findings (11). If the patient’s condition is emergent or the foreign body cannot be completely removed, laparotomy may be indicated (12, 13). In this case, the fish bone was clearly located in the extraintestinal abdominal cavity space and had caused inflammatory granulation tissue formation in the pelvic space and bladder submucosa, and caused severe adhesions among the rectum, bladder, and uterus, obscuring the normal anatomy. Therefore, laparoscopic stepwise adhesiolysis was performed to remove the pelvic mass. However, the fishbone traversed three organs, making direct intact extraction extremely difficult and prone to secondary injury. Consequently, a combined approach using hysteroscopy and cystoscopy was employed for coordinated treatment, segmental removal after severing the fishbone. This minimally invasive technique resulted in excellent therapeutic outcomes with low morbidity. One-week postoperative ultrasound follow-up revealed only a scar-like area of decreased echogenicity in the uterovesical space, and one-month follow-up demonstrated the patient reported complete resolution of symptoms, including hematuria, urinary frequency, urgency, and dysuria. Her general condition was satisfactory, and repeat Abdominal-Pelvic CT revealed no abnormalities.

In summary, interdisciplinary joint analysis provides robust support for the accurate diagnosis of gastrointestinal fish bone foreign bodies, while organizing interdisciplinary collaboration tailored to individual patient circumstances is equally critical. This case exemplifies the effectiveness and feasibility of the approach encompassing multimodal joint analysis for precise diagnosis and combined endoscopic techniques for treatment, offering valuable reference experience for the management of similar cases in the future.

## Data Availability

The original contributions presented in the study are included in the article/supplementary material. Further inquiries can be directed to the corresponding author.
